# Unmanned Aircraft Systems as a Powerful Tool to Detect Fine-Scale Spatial Positioning and Interactions between Waterbirds at High-Tide Roosts

**DOI:** 10.3390/ani12080947

**Published:** 2022-04-07

**Authors:** Johan H. Funder Castenschiold, Thomas Bregnballe, Dan Bruhn, Cino Pertoldi

**Affiliations:** 1 Department of Chemistry and Bioscience, Aalborg University, Fredrik Bajers Vej 7H, 9220 Aalborg, Denmark; db@bio.aau.dk (D.B.); cp@bio.aau.dk (C.P.); 2 Department of Ecoscience, Aarhus University, C.F. Møllers Allé 8, 8000 Aarhus, Denmark; tb@ecos.au.dk

**Keywords:** inter-species interactions, competitive behavior, choice of roosting habitat, drone, spatiotemporal positioning, density distributions, flock structure, waders, ducks, geese

## Abstract

**Simple Summary:**

In the present study, we investigated surveillance with unmanned aircraft systems (UASs) as a novel and enhanced tool to detect the individual occurrences and behavioral interactions of roosting waterbirds. We used UAS-based aerial imagery to provide fine-scale density estimations to explain intra- and inter-species interactions for 10 selected waterbird species on a major roost site in the Danish Wadden Sea. Uniquely defined density distributions were detected which, to some degree, were dependent on species and species size, with smaller waders exhibiting densely packed flocks, whereas larger species showed lower densities. Multi-species flocks were observed to occur frequently (31.9%), and generally resulted in lower densities than single-species flocks for each of the species involved. Furthermore, we demonstrated that UAS aerial photos can be used to classify in situ habitats during high-tides, which facilitated the collection of precise data for the temporal habitat choice of individual species. Our work suggests that UAS-based surveys can provide access to previously hidden aspects of the ecology for the highly dynamic communities of roosting waterbirds in the non-breeding season, important for future conservational efforts.

**Abstract:**

The surveillance of behavioral interactions between individuals in bird populations is important to understand social dynamics and explain distribution patterns caused by competition for food and space. For waterbirds, little is known about interactions between individuals at high-tide roosts. In the present study, we used surveying with unmanned aircraft systems (UASs) to provide enhanced information on previously hidden aspects of the highly dynamic communities of roosting waterbirds in the non-breeding season. Fine-scale density estimations, derived from aerial photos obtained with UASs, were used as a measure to explain intra- and inter-species interactions for 10 selected waterbird species on a major roost site in the Danish Wadden Sea. Uniquely defined density distributions were detected, which, to some degree, were dependent on species and species size, with smaller waders exhibiting densely packed flocks (e.g., dunlin *Calidris alpina* and golden plover *Pluvialis apricaria*), whereas larger species, such as ducks and geese (Anatidae) exhibited lower densities. Multi-species flocks were observed to occur frequently (31.9%) and generally resulted in lower densities than single-species flocks for each of the species involved. Furthermore, it has been demonstrated that UAS aerial photos can be used both to assess positions for roosting waterbirds and to classify habitats (i.e., mudflats, vegetated areas, waterline, and flooded areas) during high-tide. This facilitated the collection of precise data for temporal habitat choices for individual species when using the studied roost site. Our study highlights UAS surveys as an effective tool to gather hitherto unobtainable data for individual occurrences of roosting waterbirds on a spatiotemporal scale.

## 1. Introduction

Knowledge of the spatiotemporal positioning and interactions of individuals in bird populations is essential for understanding important ecological dynamics and predicting population trends and status [1,2,3,4]. Interactions between individuals on both intra- and interspecies scales are important factors when considering distribution patterns and stress levels for bird populations [3,5,6].

As avian taxa, waterbirds often occur and aggregate in areas with low and open vegetation, making them accessible and obvious targets for direct surveying [2,7,8]. During the non-breeding season, migratory waterbirds typically aggregate in large and mixed-species flocks at important stopover sites along their migratory route [9,10]. Although offering several benefits, including a reduced risk of predation [11] and increased foraging success, densely packed distributions also increase competition for food and space among the individuals [5,12]. Waterbirds in single-species communities often exhibit frequent and close encounters with repeated and aggressive interference, resulting in fluctuating behavior and creating more random distributions of individuals [12,13]. In contrast, direct inter-species interactions are rarer, and consist mainly of short outbursts to display strength [6,14]. Such displays of strength are typically skewed towards smaller species, creating displacements and establishing more defined distributions of individuals. This phenomenon has been observed for *Calidris* waders, where aggressive dominance from larger species has been reported, when interacting in mixed foraging and roosting flocks [6,13]. However, information on both intra- and inter-species interactions is still lacking or non-existing for many waterbird species.

Furthermore, species-specific habitat preferences and selection by migrating waterbirds can help explain the birds’ fine-scale utilization of both foraging areas and the non-flooded areas available for roosting at high-tide [5,9,15]. Preferences for roost sites are often influenced by proximity to the feeding grounds and anti-predator behavior, which restricts possible roost locations [9,16]. For the majority of waterbird species, this fine-scale habitat selection and utilization on roosting locations is still relatively unresearched [9,17,18]. Knowledge of factors affecting and limiting individual species in their choice of roosting sites is useful for spatial planning in the coastal zone, and can provide an increased understanding of the ecology and mechanisms of a highly dynamic habitat such as high-tide roosting sites [9].

The Wadden Sea has great international importance as a crossroad for migratory and staging waterbirds [10,19]. During high-tide, huge numbers of waterbirds congregate on a few predictable non-flooded roost sites, facilitating the monitoring of these otherwise volatile populations [1,10,16]. In the Wadden Sea, surveys of high-tide roosts are traditionally conducted by ground-based observants positioned at vantage points on nearby seawalls and dunes, or carried out from above by manned aircrafts [10]. However, these methods present substantial challenges, because large and volatile amounts of waterbirds are difficult to precisely survey from a ground-level perspective, and surveys with manned aircrafts are highly resource-intensive and further pose substantial personal risks [20,21].

An alternative to these methods is the fast-emerging unmanned aircraft systems (UAS) technology, which offers collections of data with high precision on both spatial and temporal scales [1]. Monitoring with this technology, therefore, has the potential to uncover and document until-now-hidden aspects on the spatial distribution and interactions between individuals of high-tide roosting waterbird communities [22,23]. Fast becoming affordable at the consumer level and fitted with capable sensors, small, lightweight UASs now offer an advantageous alternative to more traditional direct ground-based or manned aerial surveying. Remotely piloted, surveying with UASs can be performed by flying at low altitudes and at a slow and constant pace securing data with fine-scale resolution. Further instigated by low operating cost and nearly autonomous flights, surveying is easily repeated for an increased understanding of temporal changes and the close following of population trends and status. Unlike most traditional methods relying on in situ personnel, UAS surveying provides photographic snapshots from an aerial perspective, allowing for subsequent postprocessing and reviewing of data [20,24,25].

The aim of this study was to explore the ability of UAS surveys as a novel approach to gather enhanced information on the fine-scale spatial and temporal distribution of high-tide roosting waterbirds. We investigated: (i) to what extent information derived from UAS imagery can uncover previously hidden and unexplored species-specific distribution patterns, which is exemplified by comparisons against simulated visibilities from a ground perspective; (ii) whether intra- and inter-species interactions affect density patterns; and further (iii) the importance of the temporal habitat type for distribution and densities among different species of roosting waterbirds.

## 2. Materials and Methods

### 2.1. Study Area

**Figure 1:**
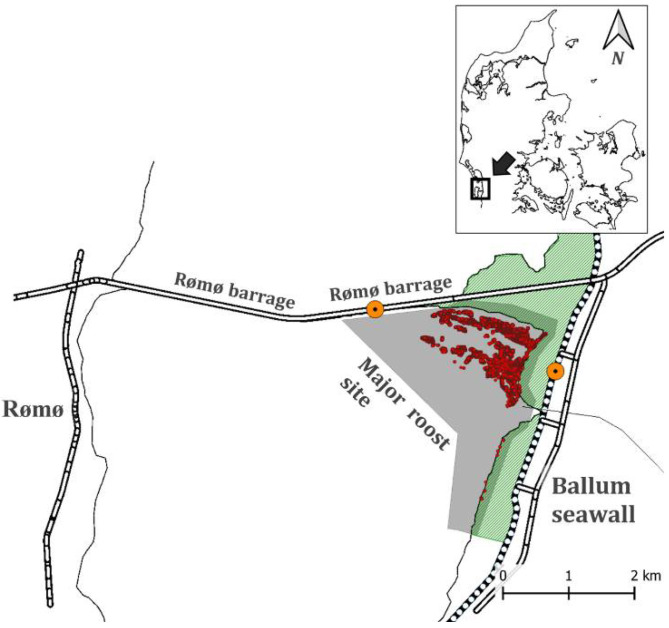
Study area in the Danish Wadden Sea. Grey shaded area indicates the investigated roost site in front of Ballum foreland. Dotted lines represent the Ballum seawall. The observation access points on the seawall are marked with orange dots. Red dots represent aggregations of roosting birds identified by UAS surveying in the study period.

The study area was located in the middle of the Danish Wadden Sea and is part of the intertidal flats stretching south of the Rømø barrage and in front of the Ballum foreland (55°14′43.30″ N, 8°67′03.80″ E) (Figure 1). These areas are considered highly important as high-tide roost sites for waterbirds. In particular, many species of waders (*Charadriiformes*), ducks (*Anatinae*), and geese (*Anserinae*) use the site during high-tide [7]. The area consisted of uniformed mudflats, and closer to the foreland, there were spots of vegetation, mainly common cordgrass (*Spartina anglica*) and common glasswort (*Salicornia europaea*). The investigated roost site area measured approximately 1.5 × 2 km^2^.

### 2.2. Field Work and Data Collection

All data used for density estimations were collected by UAS surveys on 27 September and 23 October 2019. The UAS was of the model DJI Phantom 4 Pro (DJI Technology Co. Ltd., Shenzhen, China) equipped with a 20-megapixel camera featuring 24 mm focal length [26]. The two field days (field day 1 and field day 2) were planned approximately one month apart, coinciding with the occurrence of spring tide in the area. Thus, the chance for large congregations of roosting birds was maximized. The UAS surveys were performed over a period of 4–5 h per day, covering the entire high-tide peak. This made it possible to gather information on the densities and distributions during the roosting period, and thereby for a whole tidal cycle. The target species of this study were selected to be as representative as possible for the high-tide roost, including waders (dunlin *Calidris alpina*, European golden plover *Pluvialis apricaria*, bar-tailed godwit *Limosa lapponica*, European avocet *Recurvirostra avosetta* and Eurasian oystercatcher *Haematopus ostralegus*), ducks (shelduck *Tadorna tadorna*, northern pintail *Anas acuta* and Eurasian wigeon *Mareca penelope*) and geese (greylag goose *Anser anser* and barnacle goose *Branta leucopsis*).

All UAS flights were planned and performed using the DroneDeploy (version 3.17.0, DroneDeploy, Inc., San Francisco, CA, USA) application for autopiloted surveys. The flying altitude was defined to an operating interval of 75–80 m (barometric), determined to minimize disturbance while maximizing confidence in species identification for the utilized model of UAS, DJI Phantom 4 Pro (authors’ gathered data and experiences, unpublished material). By flying at lower altitudes, behavioral alterations were provoked for several species, in particular, smaller waders (e.g., *Calidris* spp.) and geese; responses were noted to increase exponentially during lower flybys. In contrast, species of ducks (e.g., Wigeon) showed the highest tolerance to the UAS (flushing at 15–25 m). Consequently, to accommodate all target species, the altitude was kept at a minimum of 75 m. The requirements of VLOS (visual line of sight) were observed during all flights, implying several launch sites in nearby proximity of the investigated intertidal flats [27].

### 2.3. Data Preparation and Extraction

**Figure 2:**
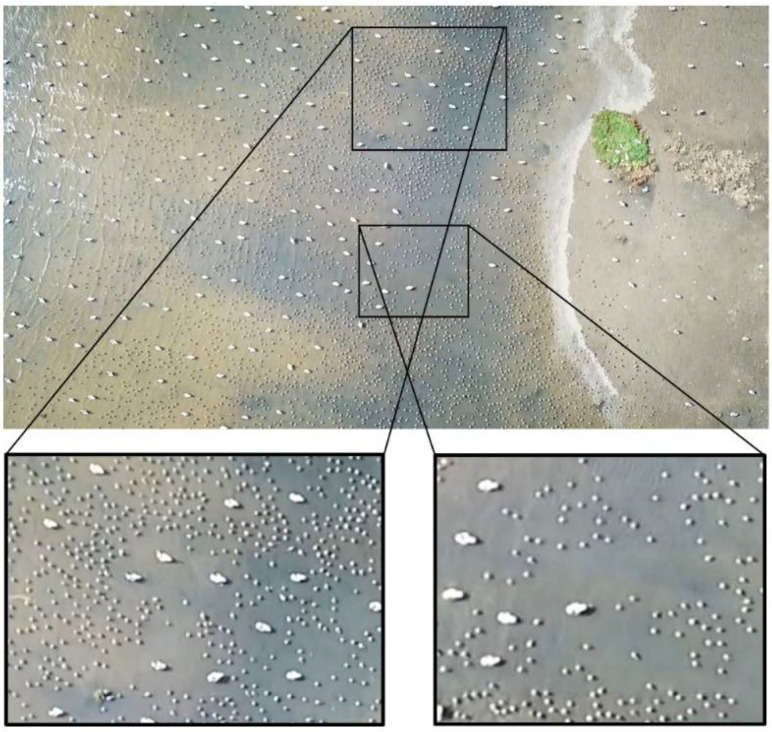
Example of an orthomosaic with two excerpts, which was generated from the UAS surveys on field day 1 (27 September). Two species, dunlin and shelduck, were present on the roost site. Precise spatial positioning was readily obtainable, which enabled the subsequent fine-scale calculation of species-specific density estimations. Note the alterations for the density distribution of dunlin, when close to the more evenly distributed individuals of Shelduck, which indicates dynamic interactions between the two species on the roost site.

Data consisting of aerial photos from the two UAS surveys were joined and stitched into Orthomosaics, using the ESRI Drone2Map for ArcGIS application (version 2.0.2, ESRI, Redlands, CA, USA), and automated georeferenced in ArcGIS Pro (version 2.5.0, ESRI, Redlands, CA, USA). Data for densities and species distribution were extracted in ArcGIS Pro by manually identifying and counting all individual spatial occurrences of birds on the georeferenced orthomosaics (Figure 2). Separate shapefile layers were created for the two field days with unique identifiers and specifications for all 10 investigated species. In total, 71,918 individual bird positions were detected on field day 1, and 71,539 individual bird positions on field day 2. The bird positions were distributed among eight species on field day 1, and 10 species on field day 2 (Figure S1).

### 2.4. Data Analysis

#### 2.4.1. Spatial Positioning from Different Viewing Perspectives

Patterns in the fine-scale spatial distribution of roosting waterbirds were explored and assessed using the identified individual spatial positions, which allowed for the calculation of species-specific distributions. To illustrate the spatial positioning from both aerial and ground viewing perspectives, two excerpts containing typical clustering of roosting birds were extracted from the UAS surveys (27 September and 23 October). By using the geo-tagged UTM (Universal Transverse Mercator) projected metric map units obtained in ArcGIS Pro, all individuals could be plotted accordingly to their spatial position. This was achieved using the R package “raster” [28]. Furthermore, density frequencies across the excerpted area were calculated, to simulate the expected view angle from ground perspective, thereby following the appearance of birds, using the R packages “ggpubr” [29] and “cowplot” [30].

#### 2.4.2. Density Patterns

Using the identified spatial positions for the individual birds allowed for the calculation of species-specific density heatmaps, which were created by applying the kernel density estimator [31]. Estimations of kernel densities were performed in ArcGIS Pro, using the “Kernel density” tool in the Spatial Analyst toolbox, and unique densities were extracted to each point with the “Extract Value to Point” tool. Initially, density estimates were assessed with boxplots (Figure S1).

Calculations of median density estimations and 95% confidence intervals (CIs) were performed for each species by bootstrapping (BCa), with 10,000 replications using the “boot” package [32] in R [33]. Next, to assess the properties of density distributions and flock structure, Shapiro–Wilk tests for normality were initially performed, following calculations of skewness by the D’Agostino test [34] and kurtosis by the Anscombe–Glynn test [35].

#### 2.4.3. Mixed and Non-Mixed Flocks

**Figure 3:**
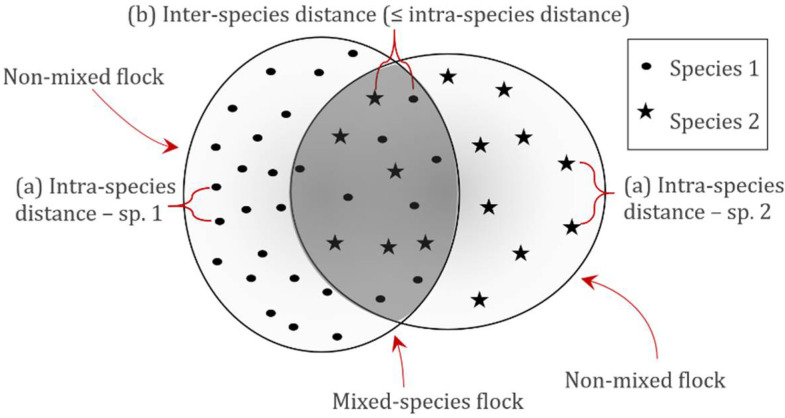
Method for differentiating between mixed and non-mixed flocks of roosting waterbirds. First, (a) intra-species distances were measured for each species. Inter-species distances (b) were then compared with the determined intra-species distances. If equal to or below the 95th percentile of these intra-species distances, the individual was considered part of a mixed-species flock.

Possible influences of species composition on densities and flock structure were investigated. To differentiate between density distributions of individuals being part of single-species (non-mixed) flocks or multi-species (mixed) flocks, specific distances between individuals of the same species (intra-species distances) were calculated for each of the 10 target species. These intra-species distances were in this study used as an objective measure for flock density. The distances between two individuals of different species (inter-species distance) could then be used to differentiate between individuals belonging to the same flock or being part of two different flocks, with the assumption that individuals with inter-species distances exceeding the intra-species distances could be considered to stem from two different flocks (Figure 3).

Intra-species distances were measured by self-joining each species-specific layer, and inter-species distances were measured by joining each species layer to all other species layers. Both measures were performed using the “Near” tool in ArcGIS Pro. Subsequently, by calculating the 95th percentiles of the intra-species-specific distances, individuals were pooled into either mixed or non-mixed flocks (Table S1). The intra-species 95th percentile distances for all species pooled were 2.4 m (field day 1) and 3.7 m (field day 2). Differences between mixed and non-mixed flocks were tested using the Wilcoxon test for matched pairs [36], and further differences in variance were tested using Levene’s test for equality of variances, which was performed for each species and generally for all individuals pooled [37].

#### 2.4.4. Inter-Species Interactions

Fine-scale spatial distributions on high-tide roosting grounds were assessed to reveal possible specific species–species interactions, observable as alterations in species-specific densities. To this end, we investigated two measures of inter-species influences: (a) distance to the nearest individual of another species; and (b) the number of individuals of all other species within a circular zone of 10 m around each individual. The 10 m circular zones were for both measures; in this study, this was assumed to be the maximum distance for detectable inter-species influences. Calculations for the nearest other species (nearest neighbor analysis) were performed in ArcGIS Pro, using the “Near” tool and total number within the circular zone of 10 m, calculated by setting the option “count” in the “Spatial Join” tool. To assess the best fit, quadratic polynomial regressions for densities against the two interaction variables were performed for each species. Data were grouped in species-specific percentile intervals of even spreading according to number of individuals, with a minimum of 5 observations in each interval, and a maximum of 20 intervals, with both parameters based on either the distance to the nearest other species (a) or individuals inside the circular zone (b). For each percentile data grouping, 95% CIs were calculated by bias-corrected and accelerated (BCa) bootstrapping with 10,000 replications, using the “boot” package in R [32]. Additionally, for each species, the determination coefficient (*R*^2^) and Spearman’s rank correlation coefficient (*R_s_*) were calculated [38].

#### 2.4.5. Habitat Selection and Density

The habitat selection of the roosting waterbirds on the roost site was assessed to determine the influence of specific habitat types on bird densities. Classifications of habitat types and distribution on the roost site were conducted for the combined UAS flights for each of the two field days. This analysis was performed in Trimble eCognition Developer (version 9.1, Trimble, Inc., Sunnyvale, CA, USA) software (Figure S2), by multiresolution segmentation analysis of the UAS-derived orthomosaics. The habitats were divided into four classes: Vegetation—vegetated areas; Mudflats—exposed intertidal flats; Waterline—intermediate zone between water and intertidal flats; and Water—water-covered areas. The environment of intertidal flats is fast-changing; therefore, the habitat classifications of the orthomosaics were derived from the same UAS survey as the bird positions, thereby matching the same spatiotemporal scale. This allowed for assessments of fine-scale in situ habitat selection at the roost site.

The habitat selection of the roosting waterbirds was explored and assessed in two ways: *(a)* all identified individuals were pooled and densities were calculated for each habitat class; and *(b)* the percentage of roosting individuals was weighted equally among the 10 investigated species for each habitat class. The formulas used, *(a)* and *(b)*, can be written as follows, where *hab[i]* denotes the habitat in question, *K_dens_* denotes the Kernel density in that habitat for all species combined and *Freq* denotes the number of occurrences of a species in the given habitat:

$$\left(a\right)Hab{\left[i\right]}_{density}=\sum K_{dens}\left(species\right)\;\;\;\left(b\right)\;Hab{\left[i\right]}_{percent}=\;\frac{Freq\left(species\right)\left[i\right]}{\sum Freq\left(species\right)}*100$$

## 3. Results

### 3.1. Spatial Positioning from Different Viewing Perspectives

**Figure 4:**
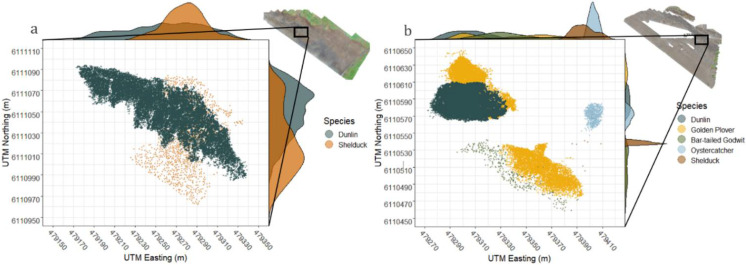
Orthomosaic excerpts from UAS fights with frequencies of density distribution. (**a**) Excerpt from 27 September and (**b**) excerpt form 23 October. The excerpts were projected in UTM metric coordinates. Both excepts contained multispecies distributions commonly encountered on the UAS-derived orthomosaics. On the *y*-axis and *x*-axis, frequency distributions are simulated, when observing the area from a ground perspective.

The two imagery excerpts from the UAS orthomosaics showed highly overlapping density distributions (Figure 4). These distributions were easily identifiable from an aerial perspective when performing UAS surveys. For comparison, when exploring the depicted distribution frequencies (Figure 4) that are visualized from a ground perspective, considerable parts of the species distributions are hidden behind each other, resulting in possible non-detectable individuals obstructed by close-standing birds.

### 3.2. Density Patterns

**Figure 5:**
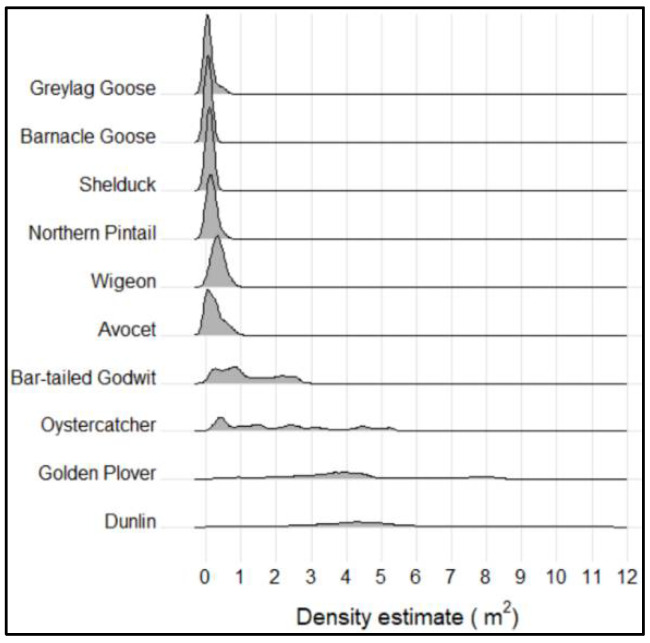
Kernel density frequencies and distributions for the 10 selected and representative high-tide roosting species investigated in this study. Densities are pooled for all individuals identified on both field days, 27 Sepptember (*n* = 71,918) and 23 October (*n* = 69,842).

The density estimations under high-tide peaks were highly dependent on the different investigated species (Figure 5). Waders showed considerably higher densities than both ducks and geese; the only exception was avocet, with a density more closely resembling the densities seen for ducks. The variations in densities were also more pronounced for waders, particularly for dunlin and golden plover, both showing kernel density frequencies in an interval from 0.01 to 11.70 (dunlin) and 0.05 to 8.60 (golden plover) individuals per square meter. In contrast, ducks and geese never showed densities exceeding 1 individual per square meter. When differentiating between the two field days, variations were detected, especially for waders (Figure S1).

Median densities under high-tide peaks were greatest for waders, in particular, dunlin (4.05 and 7.95 individuals per square meter, on the two field days, respectively) and golden glover (3.89 individuals per square meter, on the second field day) (Table 1). Avocet again stood out, with median densities (0.45 and 0.03 individuals per square meter) more similar to geese and ducks than to the other wader species. The lowest densities were observed for both species of geese and for shelduck, with the density of shelduck reaching down to ≈ 0.01 individuals per square meter. Assessing the species-specific distributions, the densities were generally positively skewed and with dispersed values (platykurtic, kurtosis ≤ 3). However, the density distribution for dunlin was significantly negatively skewed, indicating a larger proportion of the individuals standing with lower densities. In general, the densities for most species were higher on field day 2.

### 3.3. Mixed and Non-Mixed Flocks

**Figure 6:**
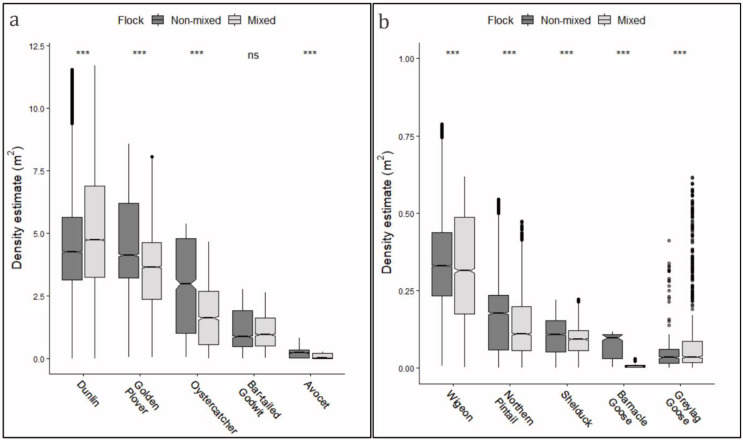
Densities for mixed (multi-species) and non-mixed (single species) flocks of roosting waterbirds, shown by boxplots. Densities were pooled for all individuals observed on both field days (27 Sepptember and 23 October). (**a**) Boxplots for density distributions for the investigated species of waders, and (**b**) boxplots for density distributions for the investigated species of ducks and geese. Differences between mixed and non-mixed flocks were tested with Wilcoxon test for matched pairs, and significance levels are depicted above each group (ns = nonsignificant, “***” < 0.001).

All 10 species had both mixed and non-mixed distributions, with an overall percentage across the investigated species of 68.1% standing in non-mixed flocks and 31.9% standing in mixed flocks. This tendency to form mixed flocks of several and varying species is clearly shown in the overlapping frequencies visualized, when viewing the roost site from ground perspective (Figure 4). In general, the median densities were higher for non-mixed single-species flocks than for mixed multi-species flocks, with high significance (*p* < 0.001, Wilcoxon test for matched pairs) (Figure 6). Simultaneously, for most species, the variance was smaller for mixed flocks than for non-mixed flocks (−9.9%; *p* < 0.001, Levene’s test), indicating a more stable density distribution for multi-species flocks. Only an opposite tendency was observed for dunlin, with both markedly higher median densities (+23%) and higher variance for mixed flocks (+53.0%; *p* < 0.001, Levene’s test). Furthermore, the variance for wigeon was also higher for mixed flocks (+15%, *p* < 0.001 Levene’s test). The tendency for greylag goose was considered non-definitive because a high number of outliers were present.

### 3.4. Inter-Species Interactions

#### 3.4.1. Distance to Nearest Other Species

**Figure 7:**
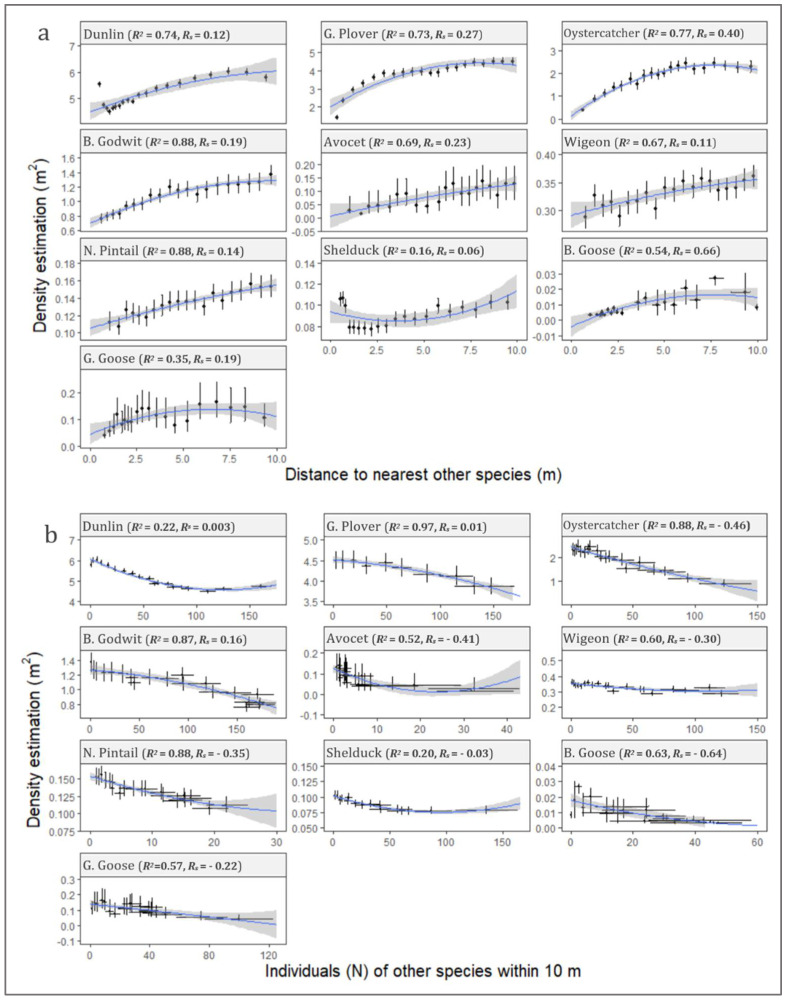
Graphical illustration of inter-species interactions expressed as altered densities. Two possible indicators for inter-species interactions were investigated: influence of nearest inter-species distance (**a**), and influence from number of individuals of other species within a 10 m circular zone (**b**). Data are grouped in percentile intervals with means and adhering 95% bootstrapped confidence intervals (CI) calculated, based on the density estimate and distance (**a**) and number (**b**). Quadratic polynomial regressions of the density estimations (denoted by blue lines) were derived for each species for both interaction indicators (**a**,**b**) and standard errors (SEs) calculated (grey-shaded areas). R-squared (*R*^2^) and Spearman’s rank correlation coefficient (*R_s_*) values are shown for each species.

Positive Spearman’s rank coefficients were observed for all of the 10 investigated species, which indicated a decreasing density with other individuals standing close by (Figure 7a). Quadratic regression between density estimates and distances to nearest other species showed robust determination coefficients (*R*^2^) up to 0.88. Furthermore, positive regression leading coefficients were observed for 9 of the 10 species (*p* < 0.05), with the only exception being shelduck. This tendency underpinned an accelerated effect on density with decreasing distance to other individuals. For waders, robust correlations were seen with *R*^2^ values for all being between 0.69 and 0.88. Shelduck (*R*^2^ = 0.16) varied markedly from the other two species of ducks, wigeon (*R*^2^ = 0.67) and northern pintail (*R*^2^ = 0.88), with a negative leading regression coefficient and pronounced lower correlation. The correlation was generally weaker for geese than the other investigated species, but still presented an *R*^2^ value between 0.35 for greylag goose and 0.54 for barnacle goose.

#### 3.4.2. Individuals of Other Species within 10 m

Quadratic regressions between density estimates and number of individuals of other species within 10 m proximity (Figure 7b) showed similarly robust *R*^2^ (0.22–0.97) to the influences caused by the nearest different species (Figure 7a). Negative Spearman’s rank coefficients were observed for 7 of the 10 investigated species, which indicated decreasing densities with an increasing number of close-standing individuals. The regression-leading coefficients were generally significant negative (*p* < 0.05); therefore, the effects on flock density were accelerated with the number of individuals of other species in the proximity. The exceptions to this tendency were two species of waders, golden plover and bar-tailed godwit, who exhibited positive leading coefficients.

### 3.5. Habitat Selection and Density

**Figure 8:**
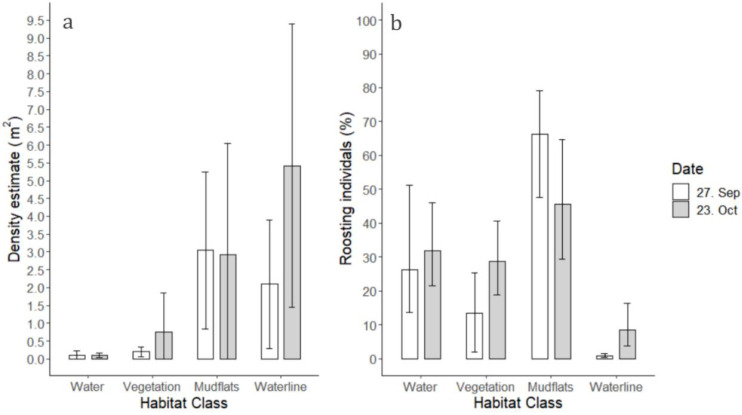
Habitat selection of high-tide roosting waterbirds in the investigated area. The available habitats on the roost site were categorized in four classes: Water, Vegetation, Mudflats and Waterline. Observations from both field days (27 September and 23 October) were differentiated in the analysis. The habitat selection was explored in two ways: (**a**) all identified individuals were pooled per flock and densities were calculated for each corresponding habitat class, and (**b**) the percentage of roosting individuals was first calculated for each species and then pooled, thereby securing an equal weighting among the 10 investigated species for each habitat class. Error bars are (**a**) represented by standard deviations (SD) of the mean and (**b**) by bootstrapped 95% confidence intervals (CIs) of the mean.

The pooled densities and distribution patterns for all species on the surveyed roost site were highly influenced by the temporal habitat (Figure 8). Of the categorized habitat variables, the highest densities (Figure 8a) were measured for Waterline (averaging 2.1 to 5.5 individuals per square meter) and Mudflats (averaging 2.9 to 3.0 individuals per square meter), whereas the habitats Water and Vegetation promoted significant lower densities (<1 individuals per square meter) (Figure S2).

When assessing quantities of roosting individuals weighted per species for the temporal habitats, Mudflats promoted the highest percentage of roosting individuals, with percentages averaging between 45% and 65% on the two field days, respectively. Waterline dropped to significantly lower frequencies than the other habitats (Figure 8b). Both the habitats of Water and Vegetation were markedly more highly represented for individuals (Figure 8b) than for species densities (Figure 8a).

Assessing habitat preferences for single species, considerable variations were seen between the two field days (Table S2). Generally, the smaller waders, golden plover and dunlin, greatly preferred the habitats of Waterline and Mudflats, with almost even distributions of 47.1% and 49.4% (field day 2), respectively, for Golden Plover, and 91.8% (Mudflats, field day 1) and 74.7% (Waterline, field day 2), respectively, for dunlin (Table S2). In contrast, the larger wader, the bar-tailed godwit, preferred Mudflats (90.8%, field day 1) and Vegetation (84.1%, field day 2). Avocet, on the other hand, had a high tendency to prefer Water habitats over Vegetation and Mudflats (58.3% and 53.5% on the two field days, respectively). Ducks were, as a group, more evenly distributed among the three habitats of *Vegetation*, *Mudflats* and Waterline. In contrast to most wader species, geese more heavily preferred the habitat of *Vegetation* together with Mudflats, especially the barnacle goose, which showed a high preference for Vegetation (field day 2).

## 4. Discussion

### 4.1. A New Perspective on Roosting Dynamics and Species Interactions

This study demonstrated that surveying with UASs allows for estimation of unique species-dependent density distributions on high-tide roosts. The possibilities found in the present study for UAS to estimate densities are in accordance with previous studies [23,39], which have noted the effectiveness of UASs as a research tool to assess densities for colony-nesting birds. Furthermore, a pilot study from 2019 [40] used a small UAS to assess behavioral traits and interactions for a family group of wild horses, with good results. However, few studies thus far have explored UASs as a research tool to investigate behavioral interactions for wildlife populations.

The high prevalence of overlapping distributions and species intermingling detected with the UAS from an aerial perspective stresses the fact that ground-based counts can be highly challenging (Figure 8). Due to the view angle, considerable degrees of species distributions may be obstructed by individuals standing in front of others, and thereby being undetected when using this traditional counting method [41,42]. The consequences of this are high degrees of over- and underestimations [22,42,43]. By deploying UAS, these challenges can be overcome and both numbers and precise individual bird positions are readily identified during postprocessing of the aerial photos. However, due to the present state of the technology, the postprocessing performed in this study was time-consuming and labor-intensive, because all individual birds had to be identified and dotted manually in GIS software. This demonstrates the need for more efficient and automated processing through the development of adaptive computer software [44,45].

### 4.2. Detailed Density Patterns

The fine-scale spatial positions derived from the aerial photos made it possible to successfully investigate the shape of the density distributions, including estimations of skewness and kurtosis for each species. The resulting density estimations showed that the distributions for the 10 investigated species were greatly influenced by species group, with waders, and particularly smaller waders, such as the dunlin and golden plover, having the highest density estimations. However, these species also exhibited the most variable distributions, both spatial, measured on the same roost site and in the same flock, and temporal, measured between the two field days.

The observed differences in density distributions were, to some extent, dependent on species body size, which could arguably be a natural explaining factor to the observed differences between the smaller waders and ducks and geese. Accordingly, one study [46] noted that the possible individual proximity of bird communities will naturally be limited by size. Of the wader species, avocet stood out by having markedly lower densities for both field days, indicating different behavioral traits for this species, when present at the roost site. An explanation to this is that avocets have longer legs, enabling the species to roost further out in deeper water [47], thereby avoiding competition from other roosting birds (Figure S3).

### 4.3. Flock Structure and Interactions

The high occurrence of multispecies flocks (31.9%), and subsequent overlapping distributions of waterbirds were observed, confirming that the mixing of species frequently occurred on high-tide roosts [7,13,42]. Simultaneously, all 10 investigated species appeared both in multi-species and single-species flocks, allowing for evaluations of characteristics for both distributions. In general, lower densities were measured for mixed flocks, together with higher variations, pointing to the fact that competitive inter-species interactions occur with ensuing decreased densities. Such interactions are accentuated by the fast-changing and dynamic environment and competition for space on the high-tide roosts [14].

Interestingly, dunlin exhibited a reversed pattern, with both higher densities and variance for mixed flocks. Explanations for this could be a different interaction strategy for dunlins, which were observed to form small but highly clumped subgroupings between the relatively dispersed individuals of larger species (Figure 2). Subsequently, the analyses performed in this study give robust indices that flock structure and density are highly influenced by both the distance to the nearest individual of another species and by the number of individuals of other species in the vicinity.

The interactions between species were visually pronounced for several species, when examining the obtained UAS aerial photos. Especially, as mentioned above, dunlin exhibited alterations for the density distribution, when close to the more evenly distributed individuals of the larger shelduck (Figure 2). The same tendency was noted by previous studies [6,13], where higher incident frequencies of inter-species aggression towards species of smaller size were observed for Calidris waders. Furthermore, one study [14] found that 97% of inter-species aggressive interactions were initiated and won by the larger species, thus establishing a strict size-related hierarchical community for mixed flocks of waders.

### 4.4. Habitat Selection

The observable highest species densities on the Mudflats and Waterline indicated general preferences for these habitats among the roosting waterbirds. Exposed mudflats were the predominately available non-flooded habitat during the tidal peak; therefore, this tendency could arguably have been expected [18,48]. Furthermore, exposed mudflats minimize the risk of unseen predators, and allow for unobstructed sight for the roosting individuals [48,49]. The Waterline, defined as the habitat of intermediate zones between water and exposed mudflats, constituted a relatively small and highly temporal area, but promoted some of the highest measured densities (averaging 5.5 [5.4; 5.7] individuals per square meter for field day 2). An explanation for this could be a closer proximity of the Waterline to the feeding grounds, making these areas highly attractive [9,48]. This preference was true for the smaller waders, dunlin and golden plover, but not exhibited by the larger waders. Both water-covered and vegetated habitats showed significantly lower densities of roosting birds, indicating a more scattered distribution in these habitats. A similar tendency of highest concentration along the immediate intertidal zone was indicated by one study [18] of the red knot Calidris canutus, by modelling data from both manual ground counts inside defined plots together with habitat composition.

When assessing the absolute number of roosting individuals on the different habitats, Mudflats still dominated, whereas Waterline became significantly less important, possibly due to its small relative area. Both Water and Vegetation habitats contained substantial numbers of birds, which was true for several species of ducks and geese. Notably, both the oystercatcher and bar-tailed godwit had high occurrences on vegetated areas on field day 2, possibly explainable by crowding of the available Mudflats. Of the waders, avocet was the only species which favored water-covered roosting habitats, with the majority of the individuals observed in this habitat. This habitat choice for avocet might correspond to the physical appearance of the species which, as mentioned previously, has long legs and is known to frequently swim during foraging [47].

## 5. Conclusions

This study showed that UASs are a powerful surveying tool for detecting individual spatiotemporal positions and species-dependent densities for high-tide roosting waterbirds. By using UASs, our study further emphasized the difficulties in observing the often-dense and multi-species aggregations occurring on high-tide roost sites, when using traditional ground-based counts.

In the present study, we uncovered strong indices for competitive interactions between the high-tide roosting species, both on intra-species and inter-species levels, which created uniquely defined density distributions and displacements. Multi-species flocks (Mixed) provoked lower densities and more defined distributions than single-species flocks (Non-mixed) for the majority of the investigated species. Furthermore, species-specific habitat preferences could be assessed in situ and on the same spatiotemporal scale, thereby counteracting inaccuracies caused by the temporal and fast-changing environment on the roost site.

To the best of our knowledge, few studies thus far have explored UASs as a research tool to investigate behavioral interactions in wildlife populations; the present study may have been the first to use UASs to perform aerial assessments of fine-scale interactions for high-tide roosting waterbirds. The data obtained by the performed UAS surveys gave a unique insight into previously hidden aspects and mechanisms for the dynamic communities of roosting waterbirds. As an exploratory study, future studies will be needed to further investigate and understand behavioral interactions in high-tide roosting waterbird communities.

## Figures and Tables

**Table 1:** Properties of the density distribution patterns for the 10 species investigated in this study measured for both field days, 27 September and 23 October. For each species, the median density (individuals per square meter) and confidence interval (CI) were calculated for the number of observed individuals (*n*). Furthermore, skewness (D’Agostino test) and kurtosis (Anscombe–Glynn test) were calculated. For both skewness and kurtosis, significance levels (sign.) are specified (ns = nonsignificant, “*” < 0.05, “**” < 0.01 and “***” < 0.001).

	27 September				23 October			
Species	*n*	Median [CI]	Skewness (Sign.)	Kurtosis (Sign.)	*n*	Median [CI]	Skewness (Sign.)	Kurtosis (Sign.)
Dunlin	49,068	4.05 [4.03; 4.06]	−0.60 (***)	3.25 (***)	21,375	7.95 [7.89; 8.01]	−0.72 (***)	2.66 (***)
Golden Plover		-	-	-	11,845	3.89 [3.85; 3.91]	0.38 (***)	2.39 (*)
Oystercatcher	1632	3.12 [3.05; 3.20]	0.12 (*)	1.81 (ns)	1286	0.57 [0.55; 0.61]	0.53 (***)	2.78 (ns)
Bar-tailed Godwit	2201	0.45 [0.43; 0.46]	0.29 (***)	1.82 (**)	5466	1.28 [1.23; 1.32]	0.09 (**)	1.95 (***)
Avocet	958	0.45 [0.42; 0.47]	0.04 (ns)	2.05 (***)	1673	0.03 [0.02; 0.04]	0.60 (***)	2.99 (ns)
Wigeon		-	-	-	25,773	0.33 [0.33; 0.33]	0.42 (***)	2.15 (***)
Northern Pintail	9491	0.17 [0.16; 0.17]	1.01 (***)	4.00 (***)	1166	0.02 [0.02; 0.03]	−1.04 (ns)	3.86 (*)
Shelduck	8005	0.10 [0.10; 0.11]	0.02 (ns)	2.28 (***)	472	0.01 [0.01; 0.01]	0.76 (***)	2.25 (**)
Barnacle Goose	55	0.01 [0.01; 0.01]	0.57 (ns)	1.62 (***)	204	0.10 [0.09; 0.10]	−0.41 (*)	3.90 (*)
Greylag Goose	508	0.03 [0.03; 0.04]	1.25 (*)	3.13 (ns)	582	0.04 [0.03; 0.04]	0.24 (*)	2.60 (ns)

## Data Availability

The data that support the findings of this study are available on request from the corresponding author (J.H.F.C.).

## Supplementary Materials

The following supporting information are available online and can be downloaded at: https://www.mdpi.com/article/10.3390/ani12080947/s1, Figure S1: Differences in species densities between the two field days; Table S1: Intra-species distances; Figure S2: Habitat types identified by Trimble eCognition software; Table S2: Species distribution frequencies for habitats; Figure S3: Roost formation of avocet.
