# Supplementary material for: Unmanned Aircraft Systems as a Powerful Tool to Detect Fine-Scale Spatial Positioning and Interactions between Waterbirds at High-Tide Roosts
Source: Animals (Basel). 2022 Apr 7;12(8):947. doi: 10.3390/ani12080947 (PMC9030221; doi:10.3390/ani12080947)
Supplement: Supplementary file 1 [file animals-12-00947-s001.zip › animals-1633060-supplementary.pdf]

Supplementary Materials

Figure S1: Differences in species densities between the two field days

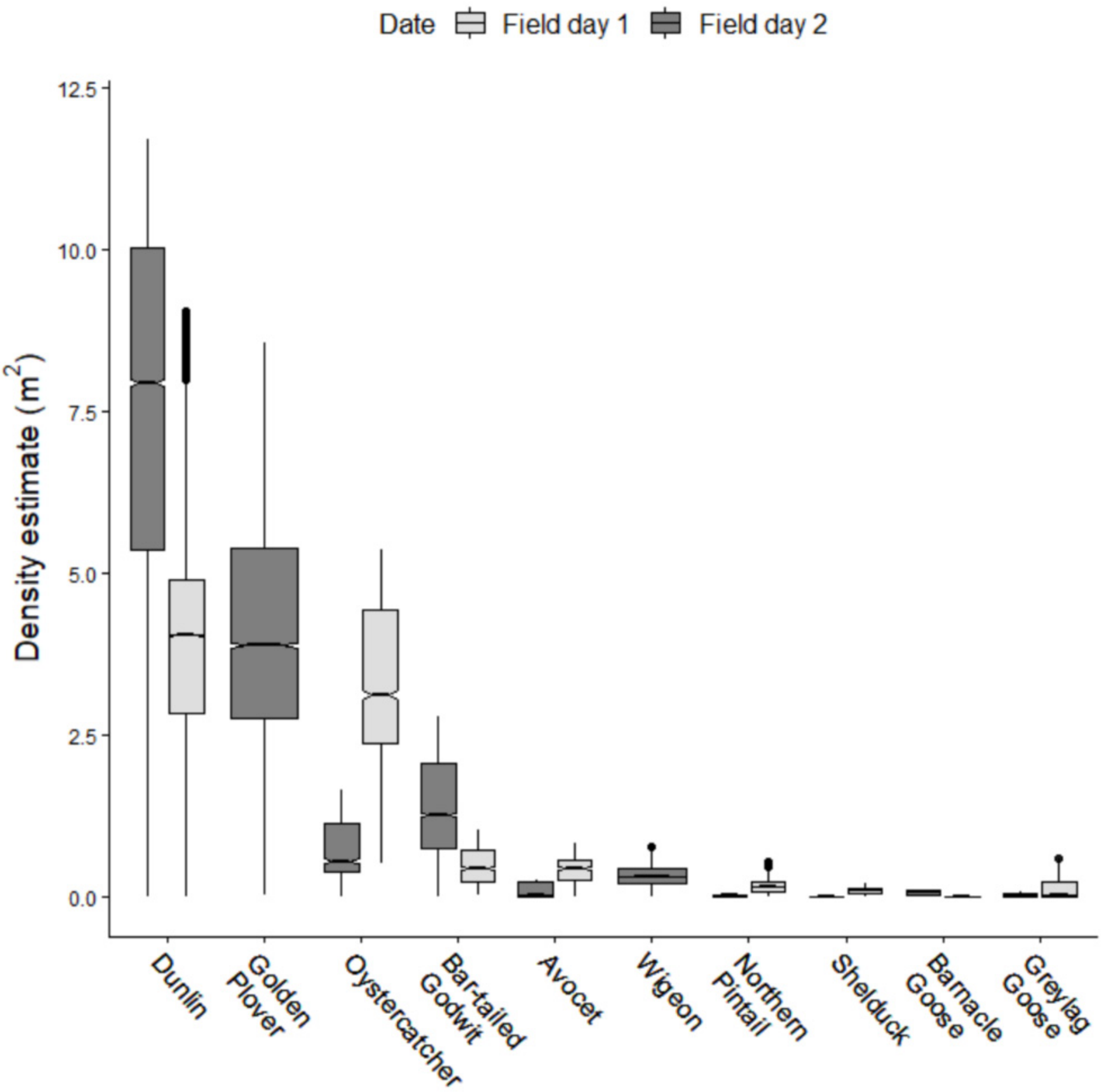

Figure S1. Density variations for the investigated species, presented by notched boxplots, observed with UAS surveys between the two field days (27. September and 23. October).

Table S1: Intra-species distances

Table S1: Intra-species distances measured for both field days, 27. September and 23. October. For each species the median (M) together with the lower 5th and upper 95th percentile are specified.

	27. September				23. October			
Species	n	5th	M	**95th**	n	5th	M	**95th**
Dunlin	49,068	0.2	0.3	**0.8**	21,375	0.12	0.2	**0.5**
Golden Plover	-	-	-	-	11,845	0.2	0.3	**0.6**
Oystercatcher	1,632	0.3	0.5	**1.1**	1,286	0.3	0.6	**1.3**
Bar-tailed Godwit	2,201	0.5	0.9	**1.9**	5,466	0.3	0.5	**1.1**
Avocet	958	0.3	0.6	**1.6**	1,673	0.4	1.1	**7.1**
Wigeon	-	-	-	-	25,773	0.3	0.6	**1.5**
Northern Pintail	9,491	0.4	0.9	**2.7**	1,166	0.6	1.2	**3.2**
Shelduck	8,005	0.8	1.5	**3.9**	472	0.9	3.0	**13.5**
Barnacle Goose	55	0.6	1.2	**5.5**	204	0.4	0.8	**3.2**
Greylag Goose	508	0.7	1.5	**3.7**	582	0.6	1.3	**4.7**

Figure S2: Habitat types identified by Trimble eCognition software

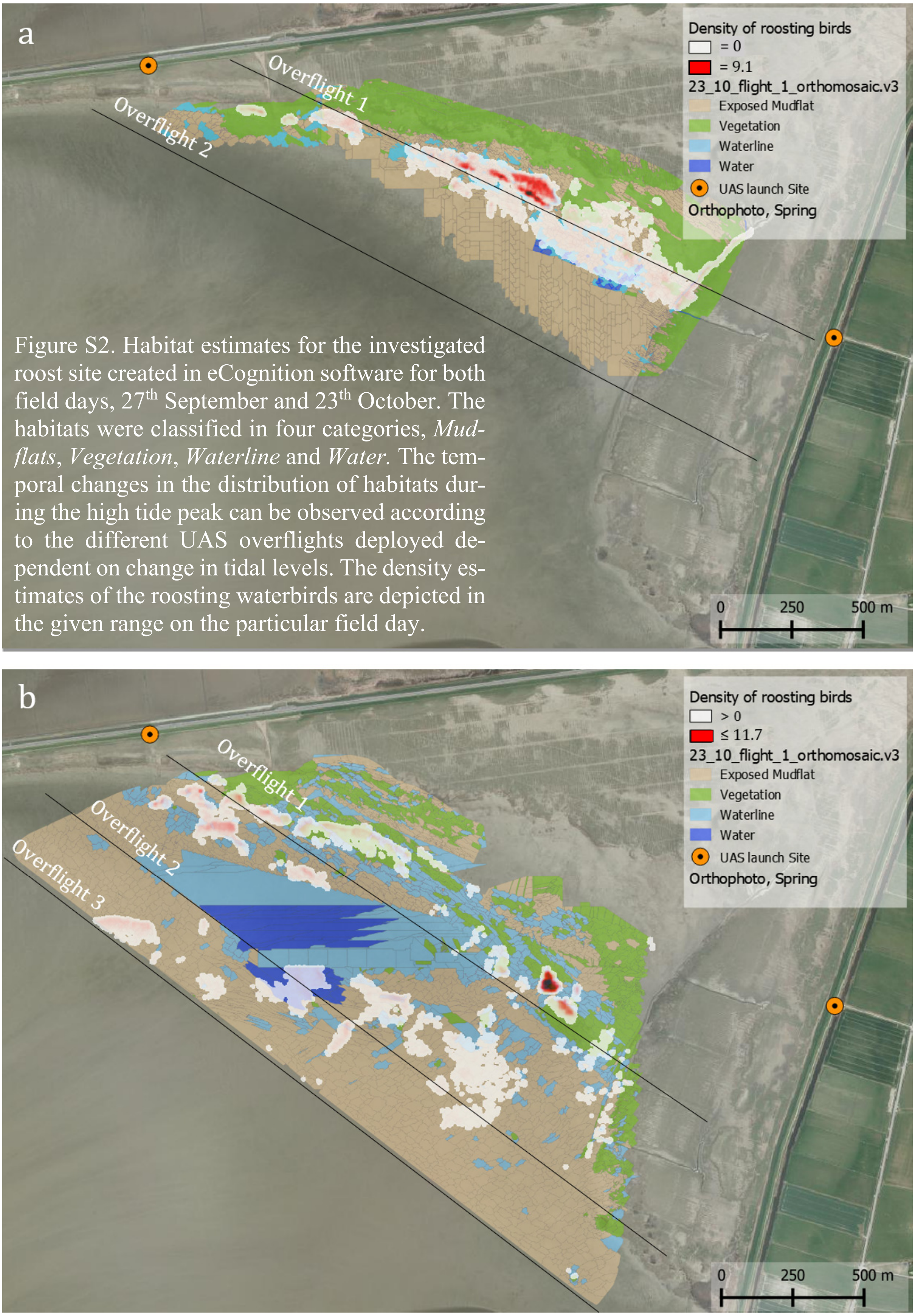

Figure S2. Habitat estimates for the investigated roost site created in eCognition software for both field days, 27th September and 23th October. The habitats were classified in four categories, *Mudflats*, *Vegetation*, *Waterline* and *Water*. The temporal changes in the distribution of habitats during the high tide peak can be observed according to the different UAS overflights deployed dependent on change in tidal levels. The density estimates of the roosting waterbirds are depicted in the given range on the particular field day.

Table S2: Species distribution frequencies for habitats

Table S2: Habitat selection for the 10 species investigated in this study measured for both field days, 27. September and 23. October. For each species the percentage (%) of individuals observed in the four habitat types is specified, *Water*, *Vegetation*, *Mudflats* and *Waterline*. The primary roosting habitat for each species on the two field days is marked (bold numbers).

	27. September					23. October				
Species	n	Water	Vegetation	Mudflats	Waterline	n	Water	Vegetation	Mudflats	Waterline
Dunlin	49,068	0.04	-	**91.8**	8.2	21,375	-	12.8	12.5	**74.7**
Golden Plover	-	-	-	-	-	11,845	-	3.5	**49.4**	**47.1**
Oystercatcher	1,632	-	-	20.7	**79,3**	1,286	-	**88.2**	11.8	-
Bar-tailed Godwit	2,201	0.9	0.1	**90.8**	8.2	5,466	-	**84.1**	2.7	13.2
Avocet	958	**58.3**	-	-	**41.7**	1,673	**53.5**	-	35.2	11.3
Wigeon	-	-	-	-	-	25,773	5.2	**52.7**	21.9	20.2
Northern Pintail	9,491	0.9	32.6	**54.8**	11.7	1,166	22.1	24.5	16.8	**36.5**
Shelduck	8,005	0.9	3.7	**81.0**	14.4	472	0.6	23.9	29.2	**46.2**
Barnacle Goose	55	1.8	-	**58.2**	40.0	204	-	**100**	-	-
Greylag Goose	508	-	17.1	**75.2**	7.7	582	6.9	13.9	**59.5**	19.8

Figure S3: Roost formation for Avocet

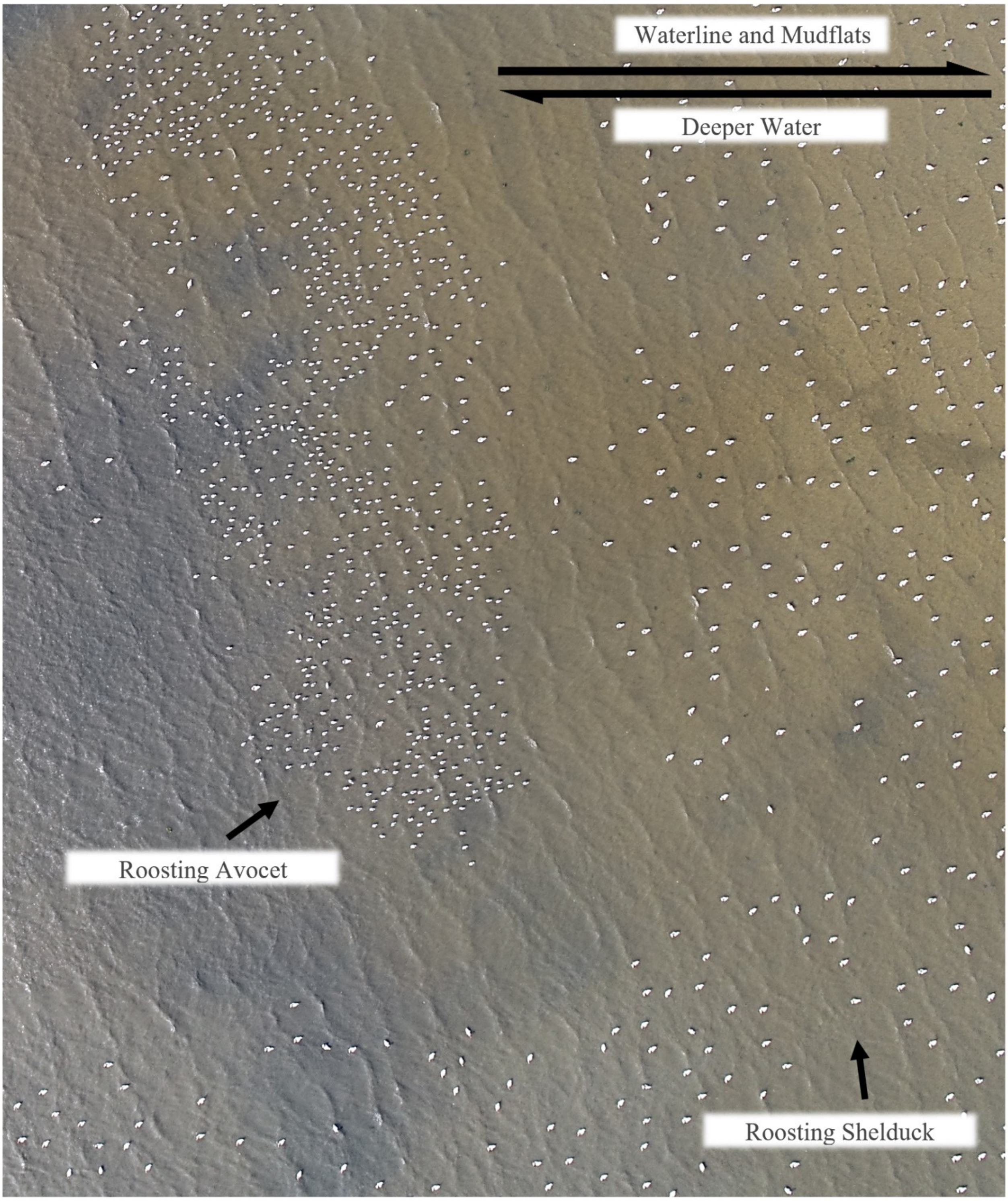

Figure S3. Roosting location and interactions between two species, Avocet and Shelduck, observed with UAS surveys on field day 1 (27. September). Note the distinct distribution for Avocet roosting further away from the exposed *Mudflats* in deeper *Water* when compared to Shelduck, thereby possibly avoiding competition from other species.
